# Lead induces the upregulation of protein arginine methyltransferase 5 possibly by its promoter demethylation

**DOI:** 10.1042/BCJ20180009_RET

**Published:** 2025-05-28

**Keywords:** DNMTs, epigenetics, lead, MEP50, PRMT5, PRMT5 promoter

This article “Lead induces the up-regulation of the protein arginine methyltransferase 5 possibly by its promoter demethylation” DOI: 10.1042/BCJ20180009 Ghosh et al., Biochem. J. 475 (16): 2653–2666 (2018) is being retracted from the *Biochemical Journal* at the request of the Editor-in-Chief and the Editorial Board. This follows receipt of a notification from a reader, alerting the Editorial Board to features in the background of Figure 3B A549 Western blot that may indicate splicing, and separately similarities in the background details between Figure 4B set 1 A549 and MCF-7 gels. Additionally, the Editorial Office identified similarities between Figure 4B Set 1 A549 UC and MC bands.

The authors were contacted regarding the concerns and provided original data for Figure 3B and Figure 4B along with a response. The Editorial Office identified signs of digital editing in the background details of the provided images. The authors’ responses and the raw data did not fully resolve the concerns raised. Therefore, the journal no longer has confidence in the reliability or validity of the results presented in this paper. Given the extent of the issues raised, the Editorial Board stand by the decision to retract the article. The authors state that the conclusion of the study is not changed and that they disagree with the decision to retract.

